# The *ATP7B* c.3316 G > A variant is associated with mild subphenotype in Wilson disease: a single-center cohort study

**DOI:** 10.1186/s13023-026-04259-9

**Published:** 2026-02-14

**Authors:** Lisheng Liu, Mingjuan Fang, Wenlong Ai, Shan Shu, Wen Zhao, Yan Yan, Nan Cheng, Wenbin Hu, Yin Xu

**Affiliations:** Institute of Neurology, Anhui University of Chinese Medicine, No. 357, Changjiang Middle Road, Hefei, 230012 China

**Keywords:** Wilson disease, Genotype-phenotype correlation, Subphenotype, Linkage disequilibrium, *ATP7B*

## Abstract

**Background:**

Genetic studies have reported the *ATP7B* c.3316 G > A variant in Wilson disease (WD). However, the phenotypic characteristics of *ATP7B* c.3316 G > A remained unclear. We aimed to explore the *ATP7B* c.3316 G > A genotype-phenotype correlation and its clinical characteristics in patients of WD.

**Methods:**

A single-center cohort study enrolled 44 WD patients with c.3316 G > A variant from 43 unrelated Chinese families (19 males [43.2%], 25 females [56.8%]), and randomly selected 44 hospitalized WD patients with *ATP7B* mutations other than c.3316 G > A as the comparison group. All phenotypic data were collected before chelation therapy. Phenotypic analyses included age of onset, clinical subtype, Kayser-Fleischer ring (KFR), copper metabolism profiles (serum copper [SCu], serum copper oxidase [SCO], ceruloplasmin [CP]), Unified Wilson’s Disease Rating Scale Part I (UWDRS-I), and Child-Turcotte-Pugh (CTP) scores. Genotype-phenotype associations were quantified in subgroup analyses.

**Results:**

The c.3316 G > A cohort harbored 21 distinct *ATP7B* mutations, including 4 nonsense, 1 frameshift, 1 synonymous, and 15 missense variants. Group analysis showed that c.3316 G > A heterozygosity showed delayed age of onset compared with non-c.3316 G > A patients (*p* < 0.001), hepatic predominance over neurological presentations (*p* < 0.001), attenuated neurological severity (UWDRS-I: *p* = 0.001), lower KFR positivity (*p* = 0.033), and relatively elevated SCu, SCO, and CP (*p* < 0.001). No gender-related phenotypic disparities were observed within either cohort. Subgroup analysis within c.3316 G > A cohort, co-mutation with- (*n* = 20) versus without- (*n* = 24) c.2333 G > T revealed no significant differences in onset age, clinical subtypes, and copper metabolism profiles. Notably, cis-arranged relationship between c.3316 G > A and c.588C > A emerged via Haploview software (v4.2) (*D’* = 1.0, LOD = 4.63, *r*^2^ = 0.239).

**Conclusion:**

The c.3316 G > A variant is associated with a distinct “mild” subphenotype characterized by delayed onset, neurologic sparing, lower KFR positivity, and milder copper dysregulation in patients of WD. Its cis-arranged relationship with the *ATP7B* c.588C > A variant provides critical insights into WD genetic diagnosis.

**Supplementary Information:**

The online version contains supplementary material available at 10.1186/s13023-026-04259-9.

## Background

Wilson disease (WD) stems from biallelic pathogenic *ATP7B* mutations that disrupt the copper-transporting P-type ATPase, impairing hepatic copper transmembrane flux [1]. This defect triggers toxic copper accumulation in the liver, brain, and other organs due to failed copper excretion. Hepatic and neurological manifestations dominate this rare disorder’s clinical spectrum, yet striking interindividual heterogeneity emerges, ranging from neuropsychiatric, renal, and osteoarticular syndromes to asymptomatic states [2]. Although *ATP7B* mutations define WD, they cannot fully account for the variations observed among different phenotypes [3]. Exploring the association between specific genotypes and clinical phenotypes remains a prominent area of interest in current WD research.

Advances in genetic diagnostics have unveiled expanding *ATP7B* mutational landscapes, providing the basis for the genotype-phenotype association [4]. For example, the c.2333 G > T variant is associated with younger age of onset and reduced ceruloplasmin(CP) levels in large Chinese cohorts [5], which is recapitulated in animal models exhibiting severe phenotypes [6]. In European populations, c.3207C > A mutations aggregate with “mild” manifestations, frequently delay symptom emergence and are associated with an increased proportion of late-onset WD [7–9]. Notably, c.3316 G > A heterozygosity has been described linking to attenuated symptomatology in two late-onset WD cases [10], with relatively elevated CP levels distinguishing carriers from other genotypes [11]. However, to date, systematic phenotyping of the c.3316 G > A variant remains absent yet.

Herein, we bridge this knowledge gap through a cohort study of 44 c.3316 G > A variant patients of WD from 43 unrelated Chinese families. This work expands the mutational architecture while decoding the variant’s phenotypic signature, ultimately delivering genetic stratification tools for precision management on WD.

## Materials and methods

### Cohort assembly

We recruited 44 hospitalized WD patients harboring the *ATP7B* c.3316 G > A variant (study group) and randomly selected 44 hospitalized WD patients with non-c.3316 G > A *ATP7B* mutations as the comparison group. The study cohort comprised patients who underwent comprehensive inpatient diagnostic assessment, including symptomatic cases and presymptomatic individuals identified through family screening or routine examination (*n* = 8). Inclusion required: (1) WD diagnosis confirmation per Leipzig criteria (score ≥4) [12, 13]; (2) complete *ATP7B* Next-Generation Sequencing (NGS) profiling with informed consent. Exclusions targeted confounders: hepatic comorbidities (viral/autoimmune hepatitis, schistosomiasis, alcoholism), neurological comorbidities (cerebral palsy, stroke, encephalitis), or incomplete full set of required baseline diagnostic assessments prior to the initiation of definitive decoppering therapy. This study received ethical approval from Medical Ethics Committee of the Affiliated Hospital of Institute of Neurology in Anhui University of Chinese Medicine with written informed consent obtained from all participants.

### Genetic characterization

All probands underwent *ATP7B* NGS with variant annotation via HGMD Pro, ClinVar, and gnomAD. Pathogenicity was classified per ACMG guidelines [14], and putative mutations were orthogonally validated through Sanger sequencing coupled with pedigree co-segregation analysis.

### Phenotypic quantification

Patients were stratified into clinical subtypes: presymptomatic (Pre), hepatic (H), neurologic (N), or mixed (H-N). Hepatic dysfunction was quantified via Child-Turcotte-Pugh scoring [15], while neurologic impairment in N/H-N patients was graded using the Unified Wilson’s Disease Rating Scale Part I (UWDRS-I) [16]. Routine biochemical assays included albumin, alanine aminotransferase (ALT), aspartate transaminase (AST), blood urea nitrogen (BUN), and creatinine (Cr), while copper metabolism-specific assays comprised ceruloplasmin (CP), serum copper (SCu), serum copper oxidase (SCO), and Basal 24-H urinary copper excretion (BUC). CP was detected using a Hitachi 7180 automatic biochemical analyzer with the turbidimetric immunoassay method. SCO was measured via the p-phenylenediamine hydrochloride colorimetric method. SCu and BUC were quantitatively analyzed by flame atomic absorption spectrometry using a WFX-120B atomic absorption spectrophotometer (Beijing Rayleigh Analytical Instrument Co., Ltd.). Two levels of internal quality control (IQC) materials (Lot No.: 1223UE and 1551UN, Randox Laboratories Ltd., UK) were run concurrently with patient samples for each batch of tests, and all results were within acceptable ranges. Reference intervals for our clinical laboratory were: SCu 10.5–22 μmol/L, and CP 200–420 mg/L. We strictly excluded patients with acute stress factors that could affect CP levels, including fever, infection, trauma, pregnancy, and other acute illnesses. KFR was documented by slit-lamp examination as an independent phenotypic indicator. All phenotypic data, including biochemical profiles, copper metabolism parameters, and KFR assessment, represent baseline measurements obtained before the start of chelation therapy.

### Genotype-phenotype interrogation

(1) c.2333 G > T interaction: Given the c.2333 G > T variant’s association with severe Asian phenotypes [5, 6], we compared the clinical profiles of patients with- and without- c.2333 G > T variant in our study group. (2) Verification of c.3316 G > A dominant effect: To decode the dominant effect of c.3316 G > A, we compared the clinical phenotype of patients with- and without- c.3316 G > A variant in the c.2333 G > T cohort. (3) Haplotype dynamics: Based on reported c.3316 G > A–c.588C > A allelic coupling [17–20], genotyping for c.3316 G > A and c.588C > A was performed in 88 WD patients. Genotype data were quality-controlled via Hardy-Weinberg equilibrium (HWE) tests (chi-square goodness-of-fit test, df = 1, *p* > 0.05). Linkage disequilibrium (LD) analysis was conducted using Haploview 4.2, with key parameters (D’, r^2^, LOD score) computed to quantify LD strength. The physical distance between variants (32150 bp) was retrieved from the UCSC Genome Browser (https://genome.ucsc.edu/).

### Statistical rigor

Statistical analyses in SPSS 23.0 were performed utilizing a Shapiro-Wilk normality test, with parametric data presented as mean±SD and analyzed using Student’s t-test, non‑parametric data as median [IQR] with Mann‑Whitney U test, categorical variables analyzed with χ^2^ or Fisher’s exact test (for binary variables) and Mann‑Whitney U test (for ordinal variables), utilizing a significance threshold set at *p* < 0.05 (two‑tailed).

## Results

### Genetic architecture of c.3316 G > A and non-c.3316 G > A cohorts

The c.3316 G > A cohort harbored 21 distinct *ATP7B* mutations, including 4 nonsense, 1 frameshift, 1 synonymous, and 15 missense variants. Among 44 patients, 42 exhibited compound heterozygous mutations (27 with 2 mutations, 13 with 3 mutations, 2 with 4 mutations), while 2 carried only one c.3316 G > A heterozygous variant (met the Leipzig WD diagnostic criteria for clinical diagnosis, but only one c.3316 G > A variant of the two alleles was successfully identified in NGS). In addition to the c.3316 G > A mutation site, the most frequent mutation was c.2333 G > T in Exon 8 (p.R778L, allelic frequency: 22.7% (20/88)), followed by c.588C > A in Exon 2 (allelic frequency: 14.8% (13/88)) exclusively in patients with ≥3 mutations (see Supplementary Table 1).

Correspondingly, the non-c.3316 G > A cohort revealed 34 mutations (5 frameshift, 3 nonsense, 2 deletions, 2 splice-site, 1 synonymous, and 21 missense), with 41 compound heterozygous mutations (37 with 2 mutations, 3 with 3 mutations, 1 with 4 mutations) and 3 homozygous mutations. Prevalent variants were c.2333 G > T in Exon 8 with allele frequency 33% (29/88, 27 patients), followed by c.2975C > T in Exon 13 with allele frequency 14.8% (13/88), and c.2612C > T in Exon 11 with allele frequency 8% (7/88) (see Table 2). A synonymous mutation, c.2310C > G, was observed in both groups and was tightly linked with c.2333 G > T. Furthermore, there were no significant differences between the groups in the types and proportions of common mutations, including missense, nonsense, and frameshift mutations.

### Phenotypic contrasts between two genotypic cohorts

In comparison to the clinical data of non-c.3316 G > A, we found that c.3316 G > A heterozygosity demonstrated: (1) Later symptom onset: median 37.5 years (IQR 19.25,47.75) vs 14 years (IQR 10,19) in Given the c.2333 G > T s (*p* < 0.001); (2) Hepatic predominance: higher hepatic subtype (H) prevalence (45.5% vs 18.2%, *p* < 0.001); (3) Reduced neurologic burden: Lower neurologic subtype (N) frequency (15.9% vs 59.1%, *p* < 0.001), attenuated UWDRS-I scores in neuro-affected patients: median 17 (IQR 12.25, 24.75) vs 37 (IQR 26, 53) (*p* = 0.001); (4) Lower KFR positivity (61.4% vs 81.8%, *p* = 0.033); (5) Milder copper dysregulation: Elevated SCu: 4.35 μmol/L (IQR 3.45, 6.06) vs 2.81 (IQR 2, 3.87) (*p* < 0.001), increased SCO: 0.114 OD (IQR 0.08, 0.17) vs 0.041 (IQR 0.035, 0.059) (*p* < 0.001), and higher CP: 119 mg/L (IQR 92.55, 143.3) vs 43.05 (IQR 36.83, 58.05) (*p* < 0.001); (6) No intergroup differences in gender distribution (19 males [43.2%] vs 27 males [61.4%], *χ*^2^ = 2.915, *p* = 0.088), ALT, AST, BUN, Cr, BUC, family history, or CTP scores (Table 1). No gender-related disparities were observed within each group (Supplementary Tables 3 , 4).

**Table 1:** Comparative clinical profiles of c.3316 G > A versus non-c.3316 G > A cohorts

Variable	c.3316 G > A (*n* = 44)	non-c.3316 G > A (*n* = 44)	Statistics	*P*
Demographics, male (%)	19 (43.2)	27 (61.4)	*χ*^2^ = 2.915	0.088
Age at onset, years	37.5 (19.25,47.75)	14 (10,19)	Z=–4.768	** < 0.001**
Subtype, n (%)			χ^2^ = 17.917	** < 0.001**
Pre	8 (18.2)	5 (11.4)		
H	20 (45.5)	8 (18.2)		
N	7 (15.9)	26 (59.1)		
H-N	9 (20.5)	5 (11.4)		
Family history, n (%)	10 (22.7)	7 (15.9)	χ^2^ = 0.656	0.418
Liver Function				
ALT, U/L	26 (16,44.75)	23 (18,42.75)	*Z*=–0.221	0.825
AST, U/L	25.5 (21,50)	30 (20,43.75)	*Z*=–0.104	0.917
Renal Markers				
BUN, mmol/L	4.91 ± 1.46	5.11 ± 1.72	t=–0.589	0.557
Cr, μmol/L	58.14 ± 20.09	59 (47.25,78.5)	*Z*=–0.931	0.352
Copper Metabolism				
BUC, μg/24 h	122.84 (73.36,268.58)†	148.57 (99.9,242.78)†	Z=–1.421	0.155
SCu, μmol/L	4.35 (3.45,6.06)	2.81 (2,3.87)	Z=–4.648	** < 0.001**
SCO, OD	0.114 (0.08,0.17)	0.041 (0.035,0.059)	Z=–6.210	** < 0.001**
CP, mg/L	119 (92.55,143.3)	43.05 (36.83,58.05)	Z=–6.547	** < 0.001**
Neurologic Assessment				
UWDRS-I§	17 (12.25,24.75)	37 (26,53)	*Z*=–3.223	**0.001**
CTP class, n (%)			*Z*=–1.109	0.267
A	35 (79.5)	39 (88.6)		
B	5 (11.4)	2 (4.5)		
C	4 (9.1)	3 (6.8)		
KFR positive, n (%)	27 (61.4)	36 (81.8)	χ^2^ = 4.526	**0.033**

Notes: Data format: Median (IQR) for non-parametric; Mean ± SD for parametric data; Significant *P*-values in bold (α = 0.05); Abbreviations: Pre: Presymptomatic subtype, H: Hepatic subtype, N: Neurologic subtype, H-N: Mixed hepatic-neurologic subtype, ALT: Alanine transaminase, AST: Aspartate transaminase, BUN: Blood urea nitrogen, Cr: Creatinine, BUC: Basal 24-H urinary copper excretion, SCu: Serum copper, CP: Ceruloplasmin, SCO: Serum copper oxidase, UWDRS-I: Unified Wilson’s Disease Rating Scale Part I, CTP: Child-Turcotte-Pugh, KFR: Kayser-Fleischer ring; Cohort specifics: †BUC sample sizes: c.3316 G > A (*n* = 36), non-c.3316 G > A (*n* = 39),§ UWDRS-I assessed exclusively in neurologic/mixed subtypes (N/H-N): c.3316 G > A (*n* = 16), non-c.3316 G > A (*n* = 31)

### c.2333 G > T phenotype showed neutrality within c.3316 G > A cohort

**Fig. 1:**
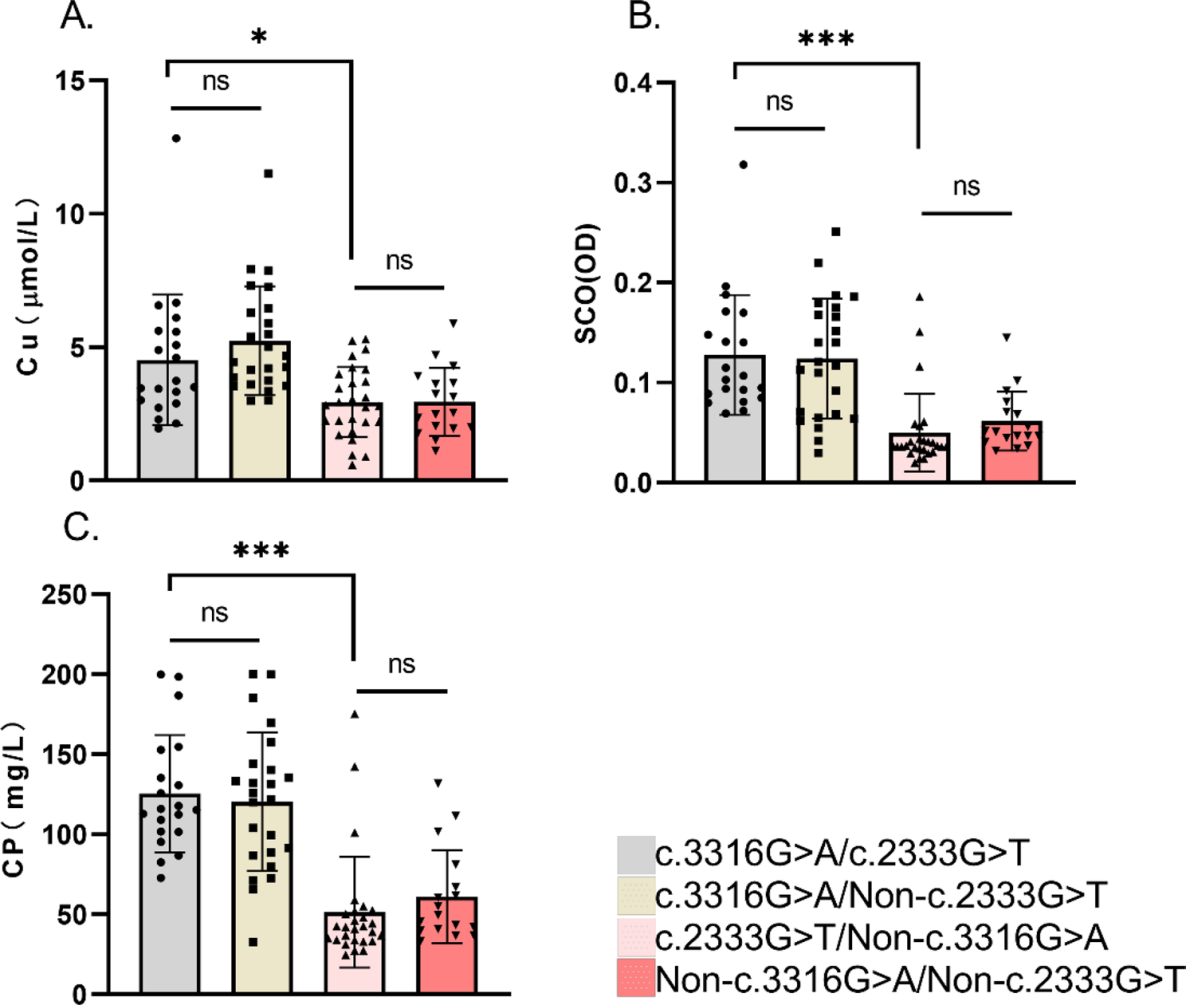
Copper metabolism in *ATP7B* subgroups. Comparative analysis of serum copper (SCu, **A**), serum copper oxidase (SCO, **B**), and ceruloplasmin (CP, **C**) levels among *ATP7B* variant subgroups. Within the c.3316 G > A cohort, no significant differences were observed between patients with (gray) or without (light yellow) the c.2333 G > T mutation. Similarly, within the non-c.3316 G > A cohort, no differences were found between patients carrying (pink) or not carrying (red) the c.2333 G > T mutation. However, patients carrying both c.2333 G > T and c.3316 G > A mutations (gray) showed significantly higher SCu, SCO, and CP levels than those carrying c.2333 G > T without c.3316 G > A (pink). Data are presented as median ± IQR, with individual points. Significance was determined by the Mann-Whitney U test (ns ˃ 0.05, *p < 0.05, **p < 0.01, ***p < 0.001). SCu, serum copper (μmol/L); SCO, serum copper oxidase (OD); CP, ceruloplasmin (mg/L)

Considering that c.2333 G > T variant is the most common mutation in Asian WD populations [5, 6], we need to exclude the influence of c.2333 G > T heterozygosity on the clinical phenotype. Subgroup analysis within c.3316 G > A cohort, co-mutation with- (*n* = 20) versus without- (*n* = 24) c.2333 G > T revealed no significant differences in onset age, clinical subtypes, family history, copper metabolism profiles (Fig. 1), CTP scores, or KFR positivity (Table 2). This indicates c.2333 G > T showed no phenotypic modulation effect in the c.3316 G > A genetic background.

**Table 2:** Phenotypic contrast of c.3316 G > A variant with- versus without- c.2333 G > T co-variants, and of c.2333 G > T variant stratified by c.3316 G > A status

Variable	c.3316 G > A + c.2333 G > T (*n* = 20)	c.3316 G > A + non-c.2333 G > T (*n* = 24)	Statistics	*P*	c.2333 G > T + c.3316 G > A (*n* = 20)	c.2333 G > T + non-c.3316 G > A (*n* = 27)	Statistics	*P*
Age at onset, years	33.55 ± 17.97	34.29 ± 16.13	t=–0.144	0.886	33.55 ± 17.97	16.56 ± 9.13	t = 4.242	** < 0.001**
Subtype, n (%)			χ^2^ = 3.249	0.355			χ^2^ = 19.446	** < 0.001**
Pre	3 (15)	5 (20.8)			3 (15.0)	2 (7.4)		
H	12 (60)	8 (33.3)			12 (60.0)	4 (14.8)		
N	2 (10)	5 (20.8)			2 (10.0)	19 (70.4)		
H-N	3 (15)	6 (25)			3 (15.0)	2 (7.4)		
Family history, n (%)	4 (20.0)	6 (25.0)	χ^2^ = 0.001	0.974	4 (20.0)	4 (14.8)	χ^2^ = 0.006	0.940
Copper Metabolism								
SCu, μmol/L	3.62 (2.93,5.61)	4.56 (3.65,6.41)	*Z*=–1.673	0.094	3.62 (2.93,5.61)	2.83 (2.19,3.99)	*Z*=–2.496	**0.013**
SCO, OD	0.105 (0.086,0.165)	0.119 (0.066,0.173)	*Z*=–0.259	0.795	0.105 (0.086,0.165)	0.037 (0.033,0.044)	*Z*=–4.865	** < 0.001**
CP, mg/L	125.43 ± 36.52	120.47 ± 43.24	t = 0.406	0.687	115.65 (101.65,148.5)	42.1 (33.8,52.2)	*Z*=–5.201	** < 0.001**
CTP class, n (%)								
A	17 (85.0)	18 (75.0)	*Z*=–0.553	0.580	17 (85)	25 (92.6)	*Z* = −0.885	0.376
B	0 (0.0)	5 (20.8)			0 (0)	1 (3.7)		
C	3 (15.0)	1 (4.2)			3 (15)	1 (3.7)		
KFR, n (%)	11 (55.0)	16 (66.7)	χ^2^ = 0.626	0.429	11 (55)	23 (85.2)	χ^2^ = 5.232	**0. 022**

Notes: Data format: Median (IQR) for non-parametric; Mean ± SD for parametric data; Abbreviations: Pre: Presymptomatic subtype, H: Hepatic subtype, N: Neurologic subtype, H-N: Mixed hepatic-neurologic subtype, BUC: Basal 24-H urinary copper excretion, SCu: Serum copper, CP: Ceruloplasmin, SCO: Serum copper oxidase, CTP: Child-Turcotte-Pugh, KFR: Kayser-Fleischer ring

### The phenotypic dominance of c.3316 G > A variant over the c.2333 G > T variant

To further validate the phenotypic dominance of the c.3316 G > A variant, we performed a subgroup analysis focusing on individuals heterozygous for c.2333 G > T. This variant is the predominant severe-phenotype mutation in Asia [5, 6]. Among patients harboring c.2333 G > T, those with and without a c.3316 G > A variant exhibited distinct clinical characteristics. Compared with non-c.3316 G > A (*n* = 27), compound heterozygous for c.3316 G > A (*n* = 20) patients exhibited: Delayed onset: 33.55 ± 17.97 years vs 16.56 ± 9.13 years (*p* < 0.001); Hepatic predominance: Higher hepatic subtype prevalence (60% vs 14.8%, *p* < 0.001); Reduced neurologic burden (10% vs 70.4%, *p* < 0.001; UWDRS-I scores: *p* < 0.001); Lower KFR positivity (55% vs 85.2%, *p* = 0.022); Attenuated copper dysregulation: elevated SCu [3.62 μmol/L (IQR 2.93, 5.61) vs 2.83 (IQR 2.19, 3.99), *p* = 0.013], greater SCO [0.105 OD (IQR 0.086,0.165) vs 0.037 (IQR 0.033,0.044), *p* < 0.001], and higher CP [115.65 mg/L (IQR 101.65,148.5) vs 42.1 (IQR 33.8,52.2), *p* < 0.001] (Fig. 1). Family history and CTP scores showed no intergroup differences (Table 2).

### c.3316 G > A – c.588C > A haplotype architecture and phenotypic characteristics

In the c.3316 G > A-positive cohort, 13 WD patients co-carried the c.588C > A mutation and harbored 3–4 *ATP7B* mutations. Family-based validation confirmed co-segregation of the two variants in all 13 families, consistent with a cis configuration. Among 88 WD patients genotyped for c.3316 G > A and c.588C > A, HWE compliance validated genotyping reliability (c.3316 G > A: *χ*^2^ = 0.00, *p* = 1.000; c.588C > A: *χ*^2^ = 0.12, *p* = 0.729). Mutant allele frequencies were 25.0% (44/176) for c.3316 G > A and 7.4% (13/176) for c.588C > A. LD analysis demonstrated complete LD between the two variants (*D*’ = 1.0, *r*^2^ = 0.24, LOD = 4.63). Recalculated LD parameters corroborated these findings, confirming a statistically significant non-random allelic association in the study cohort. Crucially, subgroup analysis within our study group *with-* (*n* = 13) versus *without-* (*n* = 31) c.588C > A, revealed no phenotypic consequences in onset age, clinical subtypes, copper metabolism, CTP scores, or KFR status (all *p* > 0.05; Table 3), demonstrating this LD lacks clinical penetrance.

**Table 3:** Phenotypic characteristics of c.588C > A co-mutation within c.3316 G > A heterozygosity

Variable	c.3316 G > A + c.588C > A (*n* = 13)	c.3316 G > A + non-c.588C > A (*n* = 31)	Statistics	*P*
Age at onset, years	33.38 ± 17.77	34.19 ± 16.66	t = −0.144	0.886
Subtype, n (%)				
Pre	1 (7.7)	7 (22.6)	χ^2^ = 5.442	0.142
H	4 (30.8)	16 (51.6)		
N	4 (30.8)	3 (9.7)		
H-N	4 (30.8)	5 (16.1)		
Copper Metabolism				
SCu, μmol/L	5.08 (3.49–6.37)	4.26(3.35,5.62)	*Z* = −0.682	0.495
SCO, OD	0.148(0.087,0.17)	0.113 (0.072–0.17)	*Z* = −0.424	0.671
CP, mg/L	120.2(98.9,137.95)	117.8 (91.6–157.7)	*Z* = −0.116	0.908
CTP class, n (%)				
A	9 (69.2)	26 (83.9)	*Z* = −1.115	0.265
B	2 (15.4)	3 (9.7)		
C	2 (15.4)	2 (6.5)		
KFR, n (%)	8 (61.5)	19 (61.3)	χ^2^ = 0.000	0.988

Notes: Data format: Median (IQR) for non-parametric; Mean ± SD for parametric data; Abbreviations: Pre: Presymptomatic subtype, H: Hepatic subtype, N: Neurologic subtype, H-N: Mixed hepatic-neurologic subtype, BUC: Basal 24-H urinary copper excretion, SCu: Serum copper, CP: Ceruloplasmin, SCO: Serum copper oxidase, CTP: Child-Turcotte-Pugh, KFR: Kayser-Fleischer ring; Statistical neutrality: All comparisons *p* > 0.05 (NS)

## Discussion

This study of 44 *ATP7B* c.3316 G > A heterozygous patients analyzed the distribution of this genotype in Chinese WD population, and revealed a distinct “mild” subphenotype characterized by delayed symptom onset, attenuated neurological manifestations (reduced UWDRS-I scores), lower KFR positivity, and milder copper dysregulation, while hepatic severity remained comparable to comparator groups. Critically, these effects were independent of c.2333 G > T co-mutation, establishing c.3316 G > A as an autonomous phenotypic modifier. Additionally, we identified a cis-arranged relationship of c.3316 G > A and c.588C > A in WD patients, exclusively on c.3316 G > A haplotypes. To our knowledge, this is the first systematic characterization of this specific genotype and its disease-modifying effects in WD.

While > 800 pathogenic *ATP7B* variants fragment WD’s genetic landscape [21, 22], their distribution exhibits strict allelic hierarchy. c.2333 G > T dominates Asian populations as the principal severity determinant [5, 23–25], whereas c.3316 G > A was once misclassified as a polymorphism [23, 24]. However, mounting evidence now confirms its pathogenicity [10, 17, 26]. Recent studies report the high prevalence of c.3316 G > A in Chinese cohorts [18, 27–29], which may suggest it as a phenotypic modulator of WD. Our cohort reveals asymmetric genetic architecture: the c.3316 G > A cohort show mutation profiles skewed toward missense variants (85.7%) with c.3316 G > A > c.2333 G > T > c.588C > A predominance, which contrasts with the c.2333 G > T-centric architecture in the non-c.3316 G > A group.

c.3316 G > A was likely to predominate in late-onset WD. As noted in existing literature (including Huster D et al.’s work on the Cu-ATPase activity–severity correlation) [30], preliminary evidence from Liu X-Q et al. [10] indicated that late-onset WD patients carrying the c.3316 G > A heterozygous variant exhibit the mildest reduction in Cu-ATPase activity among WD genotypes, alongside attenuated neurological symptoms and slow disease progression. Our observation of a mild clinical subphenotype associated with the c.3316 G > A variant aligns with the established model in WD where clinical severity correlates with the level of residual copper-transporting ATPase activity [30]. As a missense mutation, c.3316 G > A is predicted to allow for partial protein function. This retained activity would delay copper accumulation, leading to the later onset, hepatic predominance, and attenuated biochemical and neurological disturbances we documented. This functional perspective strengthens the biological plausibility of our genetic association. Early reports noted attenuated neurological progression in heterozygotes, with functional assays demonstrating superior Cu-ATPase activity preservation [10]. This aligns with genotype-phenotype correlations: CP levels inversely correlate with severity [5, 11], and c.3316 G > A cohort exhibit relatively elevated CP levels compared to the general WD population [11]. Although standard immunoassays cannot distinguish holo-/apo-ceruloplasmin [21], SCO quantification [31] confirms enzymatic preservation (*p* < 0.001). Recent genetic analyses further validate its neuroprotective role: enriched in non-neurologic WD (third most frequent variant) [32] and associated with reduced cerebral copper accumulation [33].

The c.2333 G > T exhibits severe penetrance in Asian cohorts [5, 6], associating with accelerated onset and reduced CP [34]. Wu et al. [23] compared clinical phenotypes in 18 c.2333 G > T homozygous WD patients and 11 compound heterozygotes (each with an additional *ATP7B* missense variant on the other chromosome). Relative to compound heterozygotes, c.2333 G > T homozygotes exhibited earlier onset, severe CP deficiency, and elevated mortality risk. This phenotypic difference is thought to stem from coinheritance of c.2333 G > T with other mild *ATP7B* missense variants—alone insufficiently pathogenic to induce overt WD-specific phenotypes [23]. Consistent with these clinical observations, WD mouse models harboring the c.2333 G > T variant also display early-onset neuroinflammation [6], further substantiating its pathogenic contribution to WD severity. Notably, c.2333 G > T is the second most frequent heterozygous variant among c.3316 G > A cohort. Subgroup analysis has shown no phenotypic differences in individuals with or without c.2333 G > T. However, compared to individuals carrying only c.2333 G > T, those with both c.2333 G > T and the co-mutation c.3316 G > A exhibit delayed onset, neuroprotection, and milder copper dysregulation. This suggests that the severity driven by c.2333 G > T is significantly attenuated by the presence of c.3316 G > A compared to other c.2333 G > T heterozygosity.

Typically, LD is prevalent between proximal chromosomal loci and attenuates with genetic distance, exemplified by strong LD between *ATP7B* variants c.2310C > G and c.2333 G > T (23 bp apart in exon 8) in WD [26, 27, 35]. This lineage-specific linkage is well-validated in Chinese populations: Hua et al. [27] reported c.2310C > G is rare in healthy controls (5/854) but frequent in WD patients (24/68), showing non-random association with c.2333 G > T; Qiao et al. [36] confirmed their cis configuration in South Chinese patients; Mak et al. [37] further demonstrated this linkage is exclusive to Han Chinese, with a hypothesized common ancestral origin. Notably, c.2310C > G is a synonymous variant that does not alter the encoded amino acid sequence, and no evidence supports it modulates the severe clinical phenotype driven by c.2333 G > T—its role is limited to a genetic marker for the c.2333 G > T-containing haplotype in Chinese WD cohorts.

Based on a limited cohort, our observation of LD between c.3316 G > A and c.588C > A is consistent with a recent newborn screening study [38], which found these variants in cis arrangement on a common haplotype in Chinese individuals. While neither variant has been definitively established as a mutational hotspot, their recurrent co-occurrence likely reflects a founder effect or shared ancestral haplotype in this population, rather than independent mutational events. All 13 families carrying this cis-linked haplotype were of East/North Chinese ancestry, supporting the existence of a distinct founder lineage that is separate from the well-characterized c.2310C > G/c.2333 G > T haplotype [37]. These findings advance understanding of WD’s genetic architecture and have clinical relevance: in high-risk Chinese populations, the LD pattern enables cost-effective screening, obviating supplementary c.588C > A testing in c.3316 G > A-positive patients.

Regarding the pathogenicity of c.588C > A, per ACMG guidelines [14], it meets criteria for Likely Pathogenic (PM2_Supporting + PM3) based on population frequency characteristics and clinical co-occurrence evidence. However, no direct evidence—including homozygous clinical cases, in vitro functional assays, or in vivo model validation—confirms its definitive pathogenicity, nor do clinical observations or population screening data support it as an *ATP7B* mutational hotspot. To date, there is no direct clinical or functional evidence establishing c.588C > A as an independent pathogenic variant. In our cohort, we did not observe a significant phenotypic difference between c.3316 G > A carriers with versus without this linked variant. While this suggests that c.588C > A may not be a major phenotypic modulator within the context of this specific haplotype, its biological role remains uncertain. The mild subphenotype we describe is therefore most confidently attributed to the c.3316 G > A missense change, which is predicted to retain partial function. The contribution of c.588C > A, if any, requires clarification through functional assays and identification of homozygous cases. In our cohort, c.588C > A variant’s co-occurrence with multiple other *ATP7B* variants (including the mild-modifying c.3316 G > A) may mask its potential pathogenic effects, precluding assessment of its standalone phenotypic impact.

Several limitations are included in this study: A) The single-center retrospective design limits longitudinal analysis; B) Due to the non-randomized cohort design and sample size constraints, results of the linkage analysis should be interpreted with caution; C) Residual copper ATPase activity testing was not performed due to experimental condition limitations; D) The absence of c.3316 G > A homozygous cases, likely due to the rarity of this genotype, is another limitation. Future studies should: (1) measure residual Cu-ATPase activity in peripheral blood cells or liver tissues from c.3316 G > A patients to quantify its correlation with clinical severity metrics; (2) expand the cohort through multi-center collaboration to screen for potential c.3316 G > A homozygous patients and consolidate these conclusions.

## Conclusion

c.3316 G > A variant is associated with a distinct “mild” subphenotype characterized by delayed onset, neurologic sparing, and milder copper dysregulation in patients of WD. Its cis-arranged relationship with the *ATP7B* c.588C > A variant provides critical insights into WD genetic diagnosis.

## Electronic supplementary material

Below is the link to the electronic supplementary material.

Supplementary material 1

## Data Availability

The datasets used and analyzed during the current study are available from the corresponding author on reasonable request.

## Abbreviations

WD: Wilson disease
KFR: Kayser-Fleischer ring
SCu: serum copper
SCO: serum ceruloplasmin oxidase
CP: ceruloplasmin
UWDRS-I: Unified Wilson’s Disease Rating Scale Part I
CTP: Child-Turcotte-Pugh
NGS: Next-Generation Sequencing
HGMD: The Human Gene Mutation Database
ACMG: The American College of Medical Genetics and Genomics
Pre: Presymptomatic subtype
H: Hepatic subtype
N: Neurologic subtype
H-N: Hepatic- neurologic subtype
ALT: alanine aminotransferase
AST: aspartate transaminase
BUN: blood urea nitrogen
Cr: creatinine
BUC: Basal 24-H urinary copper excretion
LD: linkage disequilibrium
CRISPR: Clustered Regularly Interspaced Short Palindromic Repeats
CNVs: copy number variations
