# Supplementary material for: The ATP7B c.3316 G > A variant is associated with mild subphenotype in Wilson disease: a single-center cohort study
Source: Orphanet J Rare Dis. 2026 Feb 14;21:134. doi: 10.1186/s13023-026-04259-9 (PMC13059575; doi:10.1186/s13023-026-04259-9)
Supplement: Supplementary file 1 — Supplementary material 1 [file 13023_2026_4259_MOESM1_ESM.docx]

**Supplementary materials**

**Supplementary Table 1. Mutation Spectrum of *ATP7B* Gene in the c.3316G>A Cohort**

| **ID** | **Sex** | **Age (years)** | **Clinical Phenotype** | **KFR Status** | ***ATP7B* Gene Mutation(s)** |
| --- | --- | --- | --- | --- | --- |
| NO.1 | M | 29 | Pre | P | exon15:c.3316G>A:p.V1106I |
| NO.2 | F | 18 | H | N | exon15:c.3316G>A:p.V1106I |
| NO.3 | M | 17 | N | P | exon15:c.3316G>A:p.V1106I \| exon13:c.2939G>A:p.C980Y \| exon12:c.2731G>A:p.A911T \| exon2:c.588C>A:p.D196E |
| NO.4 | F | 47 | N | N | exon15:c.3316G>A:p.V1106I \| exon13:c.2969C>T:p.A990V \| exon2:c.588C>A:p.D196E |
| NO.5 | M | 5 | Pre | N | exon15:c.3316G>A:p.V1106I \| exon13:c.2975C>T:p.P992L |
| NO.6 | M | 3 | Pre | N | exon15:c.3316G>A:p.V1106I \| exon13:c.2975C>T:p.P992L |
| NO.7 | F | 26 | N | P | exon15:c.3316G>A:p.V1106I \| exon13:c.2975C>T:p.P992L |
| NO.8 | F | 43 | H | P | exon15:c.3316G>A:p.V1106I \| exon13:c.2975C>T:p.P992L |
| NO.9 | F | 41 | H | N | exon15:c.3316G>A:p.V1106I \| exon13:c.2975C>T:p.P992L |
| NO.10 | M | 55 | H | P | exon15:c.3316G>A:p.V1106I \| exon13:c.2975C>T:p.P992L |
| NO.11 | M | 54 | H-N | N | exon15:c.3316G>A:p.V1106I \| exon13:c.2975C>T:p.P992L \| exon2:c.588C>A:p.D196E |
| NO.12 | M | 23 | N | P | exon15:c.3316G>A:p.V1106I \| exon13:c.2975C>T:p.P992L \| exon2:c.588C>A:p.D196E |
| NO.13 | F | 27 | H-N | P | exon15:c.3316G>A:p.V1106I \| exon13:c.3028A>G:p.K1010E |
| NO.14 | M | 57 | H | P | exon15:c.3316G>A:p.V1106I \| exon15:c.3263T>A:p.L1088X |
| NO.15 | M | 43 | H-N | P | exon15:c.3316G>A:p.V1106I \| exon15:c.3295G>T:p.G1099C \| exon2:c.588C>A:p.D196E |
| NO.16 | M | 18 | Pre | N | exon15:c.3316G>A:p.V1106I \| exon16:c.3551T>C:p.I1184T \| exon8:c.2327T>C:p.L776P |
| NO.17 | F | 33 | Pre | P | exon15:c.3316G>A:p.V1106I \| exon18:c.3809A>G:p.N1270S |
| NO.18 | F | 44 | H | P | exon15:c.3316G>A:p.V1106I \| exon18:c.3809A>G:p.N1270S |
| NO.19 | F | 48 | H-N | P | exon15:c.3316G>A:p.V1106I \| exon19:c.3955C>T:p.R1319X |
| NO.20 | M | 39 | H-N | P | exon15:c.3316G>A:p.V1106I \| exon19:c.3964C>T:p.R1322C \| exon12:c.2755C>G:p.R919G \| exon2:c.588C>A:p.D196E |
| NO.21 | F | 49 | H | P | exon15:c.3316G>A:p.V1106I \| exon2:c.525dup:p.Val176fs \| exon2:c.588C>A:p.D196E |
| NO.22 | M | 12 | N | P | exon15:c.3316G>A:p.V1106I \| exon20:c.4114C>T:p.Q1372X \| exon2:c.588C>A:p.D196E |
| NO.23 | F | 47 | H | N | exon15:c.3316G>A:p.V1106I \| exon8:c.2132G>A:p.G711E |
| NO.24 | F | 28 | H | P | exon15:c.3316G>A:p.V1106I \| exon8:c.2333G>T:p.R778L |
| NO.25 | F | 47 | H | P | exon15:c.3316G>A:p.V1106I \| exon8:c.2333G>T:p.R778L |
| NO.26 | F | 37 | H | N | exon15:c.3316G>A:p.V1106I \| exon8:c.2333G>T:p.R778L |
| NO.27 | M | 29 | H | N | exon15:c.3316G>A:p.V1106I \| exon8:c.2333G>T:p.R778L |
| NO.28 | M | 8 | Pre | N | exon15:c.3316G>A:p.V1106I \| exon8:c.2333G>T:p.R778L |
| NO.29 | M | 56 | H | N | exon15:c.3316G>A:p.V1106I \| exon8:c.2333G>T:p.R778L |
| NO.30 | F | 51 | H | P | exon15:c.3316G>A:p.V1106I \| exon8:c.2333G>T:p.R778L |
| NO.31 | M | 38 | H-N | P | exon15:c.3316G>A:p.V1106I \| exon8:c.2333G>T:p.R778L |
| NO.32 | M | 29 | N | P | exon15:c.3316G>A:p.V1106I \| exon8:c.2333G>T:p.R778L |
| NO.33 | F | 8 | H | N | exon15:c.3316G>A:p.V1106I \| exon8:c.2333G>T:p.R778L |
| NO.34 | F | 61 | H-N | P | exon15:c.3316G>A:p.V1106I \| exon8:c.2333G>T:p.R778L |
| NO.35 | F | 38 | N | P | exon15:c.3316G>A:p.V1106I \| exon8:c.2333G>T:p.R778L |
| NO.36 | F | 37 | H | P | exon15:c.3316G>A:p.V1106I \| exon8:c.2333G>T:p.R778L |
| NO.37 | F | 54 | H | P | exon15:c.3316G>A:p.V1106I \| exon8:c.2333G>T:p.R778L |
| NO.38 | M | 3 | Pre | N | exon15:c.3316G>A:p.V1106I \| exon8:c.2333G>T:p.R778L \| exon2:c.588C>A:p.D196E |
| NO.39 | F | 53 | H | N | exon15:c.3316G>A:p.V1106I \| exon8:c.2333G>T:p.R778L \| exon2:c.588C>A:p.D196E |
| NO.40 | M | 48 | H | P | exon15:c.3316G>A:p.V1106I \| exon8:c.2333G>T:p.R778L \| exon2:c.588C>A:p.D196E |
| NO.41 | F | 34 | H | N | exon15:c.3316G>A:p.V1106I \| exon8:c.2333G>T:p.R778L \| exon2:c.588C>A:p.D196E |
| NO.42 | F | 12 | H-N | P | exon15:c.3316G>A:p.V1106I \| exon8:c.2333G>T:p.R778L \| exon2:c.588C>A:p.D196E |
| NO.43 | F | 7 | Pre | N | exon15:c.3316G>A:p.V1106I \| exon8:c.2333G>T:p.R778L \| exon8:c.2310C>G:p.L770L |
| NO.44 | F | 55 | H-N | P | exon15:c.3316G>A:p.V1106I \| exon8:c.2336G>A:p.W779X |

**Abbreviations**: F: Female, M: Male, Pre: Presymptomatic subtype, H: Hepatic subtype, N: Neurologic subtype, H-N: Mixed hepatic-neurologic subtype, KFR: Kayser-Fleischer ring; P: Positive, N: Negative

**Supplementary Table 2. Mutation Spectrum of *ATP7B* Gene in the non-c.3316G>A Cohort**

| **ID** | **Sex** | **Age (years)** | **Clinical Phenotype** | **KFR Status** | ***ATP7B* Gene Mutation(s)** |
| --- | --- | --- | --- | --- | --- |
| NO.1 | M | 12 | H-N | P | exon8:c.2333G>T:p.R778L（Homozygous） |
| NO.2 | M | 10 | N | P | exon8:c.2333G>T:p.R778L（Homozygous） |
| NO.3 | F | 6 | H | N | exon8:c.2333G>T:p.R778L \| exon18:c.3884C>T:p.A1295V |
| NO.4 | M | 8 | Pre | N | exon8:c.2333G>T:p.R778L \| exon8:c.2310C>G:p.L770L \| exon5 c.1708-1G>C \| exon2:c.1168A>G:p.I390V |
| NO.5 | F | 8 | H | P | exon8:c.2333G>T:p.R778L \| exon8:c.2310C>G:p.L770L \| exon3：c.1369C＞T:p.Q457X |
| NO.6 | M | 16 | N | P | exon8:c.2333G>T:p.R778L \| exon8:c.2310C>G:p.L770L \| exon18:c.3809A>G:p.N1270S |
| NO.7 | M | 10 | N | P | exon8:c.2333G>T:p.R778L \| exon20:c.4114C>T:p.Q1372X |
| NO.8 | M | 16 | N | P | exon8:c.2333G>T:p.R778L \| exon2:c.525dup:p.V176Sfs |
| NO.9 | M | 14 | N | P | exon8:c.2333G>T:p.R778L \| exon18:c.3824T>C:p.L1275S |
| NO.10 | M | 14 | H | N | exon8:c.2333G>T:p.R778L \| exon18:c.3809A>G:p.N1270S |
| NO.11 | M | 20 | N | P | exon8:c.2333G>T:p.R778L \| exon18:c.3809A>G:p.N1270S |
| NO.12 | M | 14 | N | P | exon8:c.2333G>T:p.R778L \| exon18:c.3700del:p.V1234Lfs* |
| NO.13 | M | 16 | N | P | exon8:c.2333G>T:p.R778L \| exon17:c.3646G>A:p.V1216M |
| NO.14 | F | 9 | N | P | exon8:c.2333G>T:p.R778L \| exon16:c.3491_3492delAT:p.D1164Gfs*3 |
| NO.15 | M | 17 | N | P | exon8:c.2333G>T:p.R778L \| exon14:c.3532A>C:p.T1178P |
| NO.16 | M | 24 | N | P | exon8:c.2333G>T:p.R778L \| exon13:c.3056A＞T:p.H1019L |
| NO.17 | M | 15 | N | P | exon8:c.2333G>T:p.R778L \| exon13:c.2975C>T:p.P992L |
| NO.18 | M | 27 | N | P | exon8:c.2333G>T:p.R778L \| exon13:c.2975C>T:p.P992L |
| NO.19 | M | 10 | N | P | exon8:c.2333G>T:p.R778L \| exon13:c.2975C>T:p.P992L |
| NO.20 | M | 14 | N | P | exon8:c.2333G>T:p.R778L \| exon13:c.2975C>T:p.P992L |
| NO.21 | M | 18 | Pre | N | exon8:c.2333G>T:p.R778L \| exon13:c.2975C>T:p.P992L |
| NO.22 | M | 10 | N | P | exon8:c.2333G>T:p.R778L \| exon13:c.2975C>T:p.P992L |
| NO.23 | M | 12 | N | P | exon8:c.2333G>T:p.R778L \| exon11:c.2730+1G>A |
| NO.24 | F | 41 | N | P | exon8:c.2333G>T:p.R778L \| exon11:c.2621C>T:p.A874V |
| NO.25 | F | 40 | H-N | P | exon8:c.2333G>T:p.R778L \| exon11:c.2621C>T:p.A874V |
| NO.26 | F | 33 | H | P | exon8:c.2333G>T:p.R778L \| exon11:c.2621C>T:p.A874V |
| NO.27 | F | 13 | N | P | exon8:c.2333G>T:p.R778L \| c.3221C>T:p.A1074V \| exon18:c.3809A>G:p.N1270S |
| NO.28 | M | 8 | H-N | P | exon8:c.2304dup:p.M769fs \| exon12：c.2790_2792del:p.I930del |
| NO.29 | M | 10 | H | N | exon16:c.3532A>G:p.T1178A \| exon13:c.2939G>A:p.C980Y |
| NO.30 | M | 7 | H-N | P | exon13:c.3029A>C:p.K1010T \| exon3:c.1403-1416del |
| NO.31 | M | 19 | N | P | exon13:c.2975C>T:p.P992L \| exon7:c.2120A>G:p.Q707R |
| NO.32 | F | 12 | N | P | exon13:c.2975C>T:p.P992L \| exon2:c.525dup:p.V176Sfs |
| NO.33 | F | 11 | N | P | exon13:c.2975C>T:p.P992L \| exon2:c.1162C>G:p.Q388E |
| NO.34 | F | 15 | H | P | exon13:c.2975C>T:p.P992L \| exon18:c.3884C>T:p.A1295V |
| NO.35 | F | 9 | Pre | N | exon13:c.2975C>T:p.P992L \| exon13:c.2939G>A:p.C980Y |
| NO.36 | F | 16 | N | P | exon13:c.2975C>T:p.P992L \| exon13:c.3007G＞A:p. A1003T |
| NO.37 | F | 13 | N | P | exon13:c.2930C>T:p.T977M（Homozygous） |
| NO.38 | F | 26 | N | P | exon12:c.2755C＞G:p.R919G \| exon20:c.4114C>T:p.Q1372X |
| NO.39 | F | 26 | H-N | P | exon12:c.2755C＞G:p.R919G \| exon2:c.280G＞T:p.E94T |
| NO.40 | M | 5 | Pre | N | exon12:c.2755C＞G:p.R919G \| exon12:c.2804C>T:p.T935M |
| NO.41 | F | 28 | H | P | exon11:c.2621C>T:p.A874V \| exon8:c.2145C＞A:pY715X |
| NO.42 | M | 7 | Pre | N | exon11:c.2621C>T:p.A874V \| exon7:c.2111C＞T:p.T704I |
| NO.43 | M | 19 | H | P | exon11:c.2621C>T:p.A874V \| exon18:c.3809A>G:p.N1270S |
| NO.44 | F | 33 | N | P | exon11:c.2621C>T:p.A874V \| exon13:c.2975C>T:p.P992L |

**Abbreviations**: F: Female, M: Male, Pre: Presymptomatic subtype, H: Hepatic subtype, N: Neurologic subtype, H-N: Mixed hepatic-neurologic subtype, KFR: Kayser-Fleischer ring; P: Positive, N: Negative

**Supplementary Table 3. Comparison of Clinical Phenotypes Among Different Genders in the c.3316G>A Cohort**

| Variable | Male (n=19) | Female (n=25) | Statistics | *P* |
| --- | --- | --- | --- | --- |
| Age at onset, years | 29.63 ± 18.79 | 37.24 ± 14.64 | t=–1.511 | 0.138 |
| Subtype, n (%) |  |  | χ²=161 | 0.688 |
| Pre | 6（31.6） | 2（8） |  |  |
| H | 5（26.3） | 15（60） |  |  |
| N | 4（21.1） | 3（12） |  |  |
| H-N | 4（21.1） | 9（20） |  |  |
| Family history, n (%) | 5 (26.3) | 6 (20) | χ²=0.017 | 0.895 |
| Copper Metabolism | | | | |
| SCu, μmol/L | 4.2 (3.33,5.58) | 4.61 (3.51,6.34) | Z=–0.628 | 0.530 |
| SCO, OD | 0.095 (0.064,0.148) | 0.121 (0.087,0.184) | Z=–1.908 | 0.056 |
| CP, mg/L | 112.8 (79.8,135.3) | 130.7 (103,177.6) | Z=–1.907 | 0.056 |
| CTP class, n (%) |  |  | Z=–0.707 | 0.479 |
| A | 16 (84.2) | 19 (76.0) |  |  |
| B | 2 (10.5) | 3 (12) |  |  |
| C | 1 (5.3) | 3 (12) |  |  |
| KFR positive, n (%) | 11 (57.9) | 15 (56.8) | χ²=0.02 | 0.888 |

**Notes: Data format:** Median (IQR) for non-parametric; Mean ± SD for parametric data; Abbreviations: Pre: Presymptomatic subtype, H: Hepatic subtype, N: Neurologic subtype, H-N: Mixed hepatic-neurologic subtype, BUC: Basal 24-H urinary copper excretion, SCu: Serum copper, CP: Ceruloplasmin, SCO: Serum copper oxidase, CTP: Child-Turcotte-Pugh, KFR: Kayser-Fleischer ring; **Statistical neutrality: All comparisons *P* > 0.05 (NS)**

**Supplementary Table 4. Comparison of Clinical Phenotypes Among Different Genders in the non-c.3316G>A Cohort**

| Variable | Male (n=27) | Female (n=17) | Statistics | *P* |
| --- | --- | --- | --- | --- |
| Age at onset, years | 13.78 ± 5.3 | 19.94 ± 11.68 | t=–2.048 | 0.054 |
| Subtype, n (%) |  |  | χ²=0.000 | 0.993 |
| Pre | 4（14.8） | 1（5.9） |  |  |
| H | 3（11.1） | 5（29.4） |  |  |
| N | 17（63） | 9（52.9） |  |  |
| H-N | 3（11.1） | 2（11.8） |  |  |
| Family history, n (%) | 4 (14.8) | 3 (17.6) | χ²=0.000 | 1.000 |
| Copper Metabolism | | | | |
| SCu, μmol/L | 2.97 ± 1.45 | 2.92 ± 0.97 | t=–0.127 | 0.899 |
| SCO, OD | 0.053 ± 0.043 | 0.057 ± 0.020 | t=–0.308 | 0.760 |
| CP, mg/L | 40.9 (34.1,50.4) | 52.2 (41.5,61.1) | Z=–1.772 | 0.076 |
| CTP class, n (%) |  |  | Z=–1.947 | 0.051 |
| A | 26 (96.3) | 13 (76.5) |  |  |
| B | 0 (0) | 2 (11.8) |  |  |
| C | 1 (3.7) | 2 (11.8) |  |  |
| KFR positive, n (%) | 21 (77.8) | 15 (88.2) | χ²=0.225 | 0.635 |

**Notes: Data format:** Median (IQR) for non-parametric; Mean ± SD for parametric data; Abbreviations: Pre: Presymptomatic subtype, H: Hepatic subtype, N: Neurologic subtype, H-N: Mixed hepatic-neurologic subtype, BUC: Basal 24-H urinary copper excretion, SCu: Serum copper, CP: Ceruloplasmin, SCO: Serum copper oxidase, CTP: Child-Turcotte-Pugh, KFR: Kayser-Fleischer ring; **Statistical neutrality: All comparisons *P* > 0.05 (NS)**
